# A Surgical Hand-Book for the Use of Practitioners and Students

**Published:** 1889-12

**Authors:** 


**A Surgical Hand-Book for the Use of Practitioners and Students.** By FRANCIS M. CAIRD, M. B., F. R. C. S. (Ed.), and CHARLES W. CATHCART, M. B., F. R. C. S. (Eng. and Ed.), Assistant Surgeons, Royal Infirmary, Edinburgh, with very numerous illustrations, 12mo, pp. xv., 262. Price, $2.50. Philadelphia: P. Blakiston, Son & Co., 1012 Walnut street. 1889.

A surprising amount of information is contained within the covers of this little hand-book. The authors begin by showing the reader how to record surgical cases. Then the treatment of patients before and after operation is considered, and many sound practical hints are to be found under this head. The chapter on anesthetics, local and general, is good. The same can be said for the succeeding chapters, which include such subjects as Antiseptics and Wound Treatment, Arrest of Hemorrhage, Emergency Cases, Minor Surgical Operations, Bandaging, Fractures, Joint Troubles, and so on. Massage and electricity are well considered. The chapter on plaster-casting is a most useful one. The book ends with an appendix, in which one finds terse and complete information concerning the microscopical examinations of secretions and discharges, lists of instruments and appliances required in various operations, other practical hints and suggestions, and various formulæ useful in surgical practice. We have nothing but praise for this little work. Symptomatology, diagnosis, and the dangers arising under certain conditions, are given in a way sure to be remembered.

The publishers have done their work well. Type and illustrations are clear, and if worth be the criterion the price is a fair one.

